# Author Correction: Calculation of external climate costs for food highlights inadequate pricing of animal products

**DOI:** 10.1038/s41467-021-22237-6

**Published:** 2021-03-31

**Authors:** Maximilian Pieper, Amelie Michalke, Tobias Gaugler

**Affiliations:** 1 Technical University of Munich (TUM), Munich, Germany; 2 University of Greifswald, Greifswald, Germany; 3 University of Augsburg, Augsburg, Germany

**Keywords:** Governance, Environmental economics, Environmental impact, Agriculture, Economics

Correction to: *Nature Communications* 10.1038/s41467-020-19474-6, published online 15 December 2020.

The original version of this Article contained an error in Fig. 1 that omitted the Euro sign in an axis label. The *y*-axis should read ‘Monetary costs [C] in €/kg Prod’. The original Article also contained an error when reporting the proportion of grazing. The text should read ‘The feed of organic dairy cows incorporates a significantly higher proportion of grazing (29.5% compared to 5.0%)’.

These have been corrected in both the PDF and HTML versions of the Article.

