# Supplementary material for: Calculation of external climate costs for food highlights inadequate pricing of animal products
Source: Nat Commun. 2020 Dec 15;11:6117. doi: 10.1038/s41467-020-19474-6 (PMC7738510; doi:10.1038/s41467-020-19474-6)
Supplement: Supplementary file 1 — Supplementary Information [file 41467_2020_19474_MOESM1_ESM.pdf]

Supplementary Information

for

Calculation of external climate costs for food highlights inadequate pricing of animal products

Pieper et al.

Supplementary Note 1

Additional information on the determination of emission-differences between conventional and organic production:

There is not a sufficient number of papers published describing the emission differences of organic and conventional production within the German agricultural landscape only. To broaden the database for following calculations we choose to include studies from a European background. Legislative circumstances describing organic farming are the same in all of Europe. Therefore, values declared as being of organic origin can be compared with each other as the rules and activities of this production practice are clearly defined within European boundaries.

As agricultural production is influenced by climatic conditions, it is to be noted, however, that factors like precipitation or solar radiation do vary between the countries in which selected studies were conducted. However, the climatic conditions within Germany's borders vary strongly as well, especially from north to south. Some regions in Germany can therefore be more closely compared to regions of Ireland, for example, some more to regions of Spain. Since our assessment is designed to describe agricultural production of Germany in general, it is sensible that the climatic differences of the underlying studies also describe the diverse climatic conditions of Germany.

Furthermore, we use the relation between the studies' reported greenhouse gas emissions of organic and conventional agriculture rather than the reported absolute values for further calculations. Climatic conditions within one study, which could result in different emission values compared to an evaluation on German ground, will likely have similar or equal impact on both the agricultural practices assessed within this one study. We therefore argue, that the ratio does not change drastically with climatic conditions. This notion is also supported by the reported values not showing particular tendencies to one or the other direction due to their origin.

Additional information on the respective studies can be found in supplementary table 1.

Supplementary Note 2

Additional information on the data gathering in GEMIS:

A closer look into the mechanisms of GEMIS shall be provided with the example of beef. In GEMIS one can sort through a wide variety of processes. For this explanation, we want to look at the process of beef-production at the stage of slaughtering. This process is labeled with the code: NG-SchlachtereiDE-Rind-2010, which already gives some indications on the properties of the process. NG stands for ‘Nahrungs- und Genussmittel’ (english: food and beverage). This is followed by an indication on the stage of production and reference country, whereby SchlachtereiDE stands for butchery in Germany. Then the actual product (Rind=beef) and the reference year (2010) are listed.

Under this code, all inputs, outputs and the corresponding emissions are listed for one functional unit of the produce (defined as 1 kg of beef-meat). Furthermore, one can read out the compounds of this data-set. In this case, it is the electricity and process-heat for the slaughtering stage, as well as the process of animal-husbandry (including all inputs necessary to raise the animal). Reading out these data-subsets reveals their respective in- and outputs, which themselves consist of even more inputs. By repeating this recursive process, one can read out ever more fine-grained compounds, the further to the beginning of the supply chain one progresses.

Additionally to that, you can find the datasets from GEMIS that we used for our study in supplementary table 2.

Supplementary Table 1

Included studies and their specifics used for the determination of the emission-difference ($D_{org/conv}$) between organic and conventional production in different countries' contexts through the application of meta-analytical methods (compare table 2 in the main text).

Source	Estimation method	Boundaries	Regional coverage[1]	Observed food category	Emisison values [kgCO₂-eq/ha] conventional	organic	difference org/conv
Plant-based							**49%**
Aguilera et al. (2015 a) [40] p. 719 data collected: N/A	LCA modelling (empiric data from interviews and other studies)	cradle to farmgate	Spain	cereals[2]	1.024	361	45%[3]
				legumes	568	232	
				field vegetables[4]	3.448	1.418	
				vegetables greenhouse	11.841	7.592	
Aguilera et al. (2015 b) [41] p. 730 data collected: 2012 (3)	LCA modelling (empiric data from interviews and other studies)	cradle to farmgate	Spain	citrus fruits[5]	6.324	1.897	49%
				fruits	2.597	1.480	
				wine	964	641	
Cooper et al. (2011) [42] p. 189 data collected: 2004-2007 (5,5)	empiric data gathered at site	direct and indirect emissions until farmgate; comparable with cradle to farmgate	Nafferton (Northern England), UK	crop rotation[6]	2.019	841	42%
Küstermann et al. (2008) [43] p. 48 data collected: 1999-2002 (7,5)	modelling (software REPRO)	direct and indirect inputs until farmgate; comparable with cradle to farmgate	Scheyern (Upper Bavaria), Germany	crop rotation[7,8]	376	263	70%
Reitmayr et al. (1995)[44] (as quoted in Stolze et al. 2000, p. 55) data collected: N/A			Germany	winter wheat	1.001	429	63%
				potatoes	1.153	958	

[1] The specific regional coverage was not stated in all studies. Locations are stated as precisely as possible.

[2] We have excluded the in underlying study (Aguilera et al. 2011a) observed food category 'rice' for this assessment as it is an irrelevant product for the assessment of the German agricultural sector.

[3] When there was more than one food category assessed in one study, we weighted them equally to not interfere with the weighting system between the studies.

[4] In GEMIS 'field vegetables' constitutes a collective term describing vegetables that are grown in the open air. This form of cultivation is in contrast to the horticultural cultivation of vegetables which uses greenhouses, foil tunnels or other artificially protected areas.

[5] We have excluded the in underlying study (Aguilera et al. 2011b) observed food categories 'subtropical fruit trees', 'tree nuts', and 'olives' as they are irrelevant products for the assessment of the German agricultural sector.

[6] Rotation includes winter wheat, potatoes, beans, cabbage, and spring/winter barley.

[7] Rotation includes potatoes, winter wheat, sunflower, winter rye, and maize.

[8] Even if sunflower is irrelevant to the assessment of the German foodstuff it is, however, crucial for the underlying crop rotation and farming processes and was therefore not excludable from assessment.

Tuomisto et al. (2012)[45] SI table S.1 data collected: 2001-2008 (7,5)	LCA modelling (data from previous studies and empirical data)	Indirect[9] and direct inputs until farmgate; comparable with cradle to farmgate	UK	winter wheat	1.772	629	36%
Animal-based							**84%**
Basset-Mens, Wertf (2005) [46] *data collected: 1996-2001 (6,5)*	LCA modelling (data from other studies)	direct and indirect inputs and effects; comparable with cradle to farmgate	France (Bretagne)	pig	4236	4022	95%
Casey, Holden (2006)[47] data collected: N/A	LCA modelling (data from questionnaires and other studies)	cradle to farmgate	Ireland	beef	5346	2302	82%
Flessa et al. (2002)[48] data collected: 1994-1998 (6)	modelling (on basis of empirical data and other studies)	direct inputs and limited[10] indirect inputs until farmgate; comparable with cradle to farmgate	Germany, Oberbayern (South)	beef/cattle	4177	3037	73%
Dairy							**63%**
Bos et al. (2007)[49] data collected: N/A	modelling (model DairyWise)	indirect and direct emissions; comparable with cradle to farmgate	Netherlands	dairy	[11]		61%
Dalgaard et al. (2006)[50] data collected: 1999 (7)	LCA modelling (based on empirical data from 2138 private farm accounts)	cradle to farmgate[12]	Denmark	dairy; sandy soil	6.335	5.459	57%
				dairy; sandy loam soil	5.803	1.669	
Haas et al. (2001)[51] data collected: 1998 (3)	LCA modelling (based on empirical data from 35 farms in the region)	direct and upstream (indirect) processes; comparable with cradle to farmgate	Germany, Allgäu (Southern Bavaria)	dairy	9.400	6.300	67%
Thomassen et al. (2008)[52] data collected: 2003 (5)	LCA modelling (based on empirical data from field studies of 10 conventional and 11 organic farms, and data of previous studies)	cradle to farmgate	Netherlands	dairy	20.598	13.405	65%

9 Tuomisto et al. (2012) and Flessa et al. (2002) explicitly state that the production of farm buildings is not considered. However, as far as it was comprehensible, all other studies have similarly not included assessment of housing production.

10 Production of fertilizer was considered; other indirect inputs for precursors like pesticides and seeds were not included as they were considered negligible; infrastructure (machines and buildings) was not included. This studies system boundaries are least in line with our assessment scope but are still comparable due to the explanation as to why certain processes were excluded.

11 As Bos et al. (2007) reports „GHG emissions per ha on the conventional dairy farms are 65% higher than on the organic model farms." (p.3). We set organic as 100% and conventional as 165%.

12 Authors refer to cradle to grave approach when introducing to the topic of LCA. They continue although with cradle to farmgate assessments of nitrogen surpluses, for example. The input data does also not include processes after farmgate. Therefore, we find this approach to be comparable with cradle to farmgate.

Supplementary Table 2

Original GEMIS data (kgGas/kgProd)

Name	name (in GEMIS)	category (in GEMIS)	production-level (in GEMIS)	Code	CO2-eq
field-vegetables	Feldgemüse	Gemüse	Anbau	AnbauFeldgemüse-DE-2010	0.0328
Tomatoes	Tomate	Gemüse	Anbau	AnbauTomate-DE-2010	0.3943
Fruit	Obst	Obst	Anbau	AnbauObst-DE-2010	0.2531
Rye	Roggen	Getreideerzeugnisse	Anbau	AnbauRoggen-DE-2010	0.2204
Wheat	Weizen-Körner	Getreideerzeugnisse	Anbau	AnbauWeizen-Körner-DE-2010	0.3757
Oat	Hafer	Getreideerzeugung	Anbau	AnbauHafer-DE-2010	0.3605
Barley	Gerste	Getreideerzeugung	Anbau	AnbauGerste-DE-2010	0.3335
Potatoes	Kartoffeln	Gemüse	Anbau	AnbauKartoffel-DE-2010	0.0648
Beans	Bohnen	Gemüse	Anbau	AnbauFeldgemüse-DE-2010	0.0328
Rapeseed	Rapsöl	Raps-Öl	NG-Herstellung	NG-HerstellungRapsöl-DE-2010	1.0192
Eggs	Eier	Eier	Tierhaltung Legehenne	TierhaltungLege henne(Ei)-DE-2010	1.1711
Broilers	Masthähnchen	Fleisch	NG-Schlachterei	NG-SchlachtereiDE-Masthähnchen-2010	13.1718
Beef	Rind	Fleisch	NG-Schlachterei	NG-SchlachtereiDE-Rind-2010	24.8637
Pork	Schwein	Fleisch	NG-Schlachterei	NG-SchlachtereiDE-Schwein-2010	5.5486
Milk	Milch	Milchprodukte	Tierhaltung Milchkuh	TierhaltungMilchkuh (Milch)-DE-2010	1.0958
