# Supplementary material for: Calculation of external climate costs for food highlights inadequate pricing of animal products
Source: Nat Commun. 2020 Dec 15;11:6117. doi: 10.1038/s41467-020-19474-6 (PMC7738510; doi:10.1038/s41467-020-19474-6)
Supplement: Supplementary file 3 — Reporting Summary [file 41467_2020_19474_MOESM3_ESM.pdf]

nature research

Corresponding author(s): Maximilian Pieper

Last updated by author(s): Oct 12, 2020

Reporting Summary

Nature Research wishes to improve the reproducibility of the work that we publish. This form provides structure for consistency and transparency in reporting. For further information on Nature Research policies, see Authors & Referees and the Editorial Policy Checklist.

Statistics

For all statistical analyses, confirm that the following items are present in the figure legend, table legend, main text, or Methods section.

n/a	Confirmed	
☒	☐	The exact sample size (*n*) for each experimental group/condition, given as a discrete number and unit of measurement
☒	☐	A statement on whether measurements were taken from distinct samples or whether the same sample was measured repeatedly
☒	☐	The statistical test(s) used AND whether they are one- or two-sided<br>*Only common tests should be described solely by name; describe more complex techniques in the Methods section.*
☒	☐	A description of all covariates tested
☒	☐	A description of any assumptions or corrections, such as tests of normality and adjustment for multiple comparisons
☒	☐	A full description of the statistical parameters including central tendency (e.g. means) or other basic estimates (e.g. regression coefficient) AND variation (e.g. standard deviation) or associated estimates of uncertainty (e.g. confidence intervals)
☒	☐	For null hypothesis testing, the test statistic (e.g. *F*, *t*, *r*) with confidence intervals, effect sizes, degrees of freedom and *P* value noted<br>*Give P values as exact values whenever suitable.*
☒	☐	For Bayesian analysis, information on the choice of priors and Markov chain Monte Carlo settings
☒	☐	For hierarchical and complex designs, identification of the appropriate level for tests and full reporting of outcomes
☒	☐	Estimates of effect sizes (e.g. Cohen's *d*, Pearson's *r*), indicating how they were calculated

Our web collection on statistics for biologists contains articles on many of the points above.

Software and code

Policy information about availability of computer code

Data collection	Emission values were derived from the publicly available material flow analysis tool GEMIS (Version 4.95)
Data analysis	Microsoft Excel (for Mac, version 16.16.26) was used to calculate and analyse the data of this study.

For manuscripts utilizing custom algorithms or software that are central to the research but not yet described in published literature, software must be made available to editors/reviewers. We strongly encourage code deposition in a community repository (e.g. GitHub). See the Nature Research guidelines for submitting code & software for further information.

Data

Policy information about availability of data

All manuscripts must include a data availability statement. This statement should provide the following information, where applicable:

- Accession codes, unique identifiers, or web links for publicly available datasets
- A list of figures that have associated raw data
- A description of any restrictions on data availability

The datasets generated and analyzed during the current study are available in the Center for Open Science repository, https://osf.io/e7v8x/?view_only=0bff6aa858a340df9046816c1404a51c. The source data underlying Table 1-4, Figure 1, Figure 2 and the Supplementary Notes are provided in this source data file. The datasets are derived from the following databases: German Federal Office of Statistics (https://www-genesis.destatis.de/genesis/online), German Society for Information on the Agricultural Market (AMI) (https://www.ami-informiert.de/), KTBL-Standard Gross Margins (https://daten.ktbl.de), EU Open Data Portal (https://data.europa.eu/euodp/en/data/dataset/uLrJZE2PQkMHod6feE8gXQ), Eurostat (https://ec.europa.eu/eurostat/databrowser), German Federal Office for Agriculture and Food (BLE) (https://www.ble.de), German Head Organization of Ecological Food Economics (BÖLW) (https://www.boelw.de/), Expert Agency for Renewable Resources (FNR) (https://fnr.de/) and the German Federal Ministry for Food and Agriculture (BMEL) (https://www.bmel-statistik.de/). More detailed information is provided in the source data file.
Microsoft Excel (for Mac, version 16.16.26) was used to calculate and analyze the data of this study.

Emission values were derived from the publicly available material flow analysis tool GEMIS (Version 4.95), which can be downloaded here: http://iinas.org/gemis-download-121.html

Field-specific reporting

Please select the one below that is the best fit for your research. If you are not sure, read the appropriate sections before making your selection.

☐ Life sciences ☐ Behavioural & social sciences ☒ Ecological, evolutionary & environmental sciences

For a reference copy of the document with all sections, see nature.com/documents/nr-reporting-summary-flat.pdf

Ecological, evolutionary & environmental sciences study design

All studies must disclose on these points even when the disclosure is negative.

Study description	Our study only involved preexisiting datasets. These include the production quantities as well as the producer prices of foodstuff produced in Germany in 2016.Furthermore, emission values for conventional foodstuff were derived from the material flow analysis tool GEMIS. To determine the emission difference between conventional and organic production we analyzed 13 different studies with meta-analytical methods.
Research sample	The data of production quantities as well as the producer prices of foodstuff was derived from the German Federal Office of Statistics (Destatis) and the Agricultural Market Information Company (AMI). Furthermore, emission values for conventional foodstuff were derived from the material flow analysis tool GEMIS. To determine the emission difference between conventional and organic production we analyzed 13 different studies with meta-analytical methods.
Sampling strategy	Since we did not collect own primary data, no 'sampling strategy' in the narrow sense was used.
Data collection	Since we did not collect own primary data, 'data collection' in the narrow sense did not take place. Instead, data from existing LCA tools were used. The identification of studies on the difference between different cultivation systems primarily took place between April 2018 and September 2018. The Identification of data / studies was expanded as part of the revision process selectively in the first half of 2020.
Timing and spatial scale	For all data used in this study, the reference year is 2016 and the regional context is Germany.
Data exclusions	In the context of our study no relevant data from the predefined regional and temporal context was excluded.
Reproducibility	Based on the data and sources made available, our results can be reproduced.
Randomization	We did not collect own primary data and did not carry out statistical tests. There were no experiments with experimental and control groups. A randomization is therefore not possible due to methodological reasons.
Blinding	We did not collect own primary data and did not carry out statistical tests. There were no experiments with an experimental group, nor with an investigator. Therefore, there was no need to take precautions to ensure blinding.

Did the study involve field work? ☐ Yes ☒ No

Reporting for specific materials, systems and methods

We require information from authors about some types of materials, experimental systems and methods used in many studies. Here, indicate whether each material, system or method listed is relevant to your study. If you are not sure if a list item applies to your research, read the appropriate section before selecting a response.

Materials & experimental systems

n/a	Involved in the study
☒	☐ Antibodies
☒	☐ Eukaryotic cell lines
☒	☐ Palaeontology
☒	☐ Animals and other organisms
☒	☐ Human research participants
☒	☐ Clinical data

Methods

n/a	Involved in the study
☒	☐ ChIP-seq
☒	☐ Flow cytometry
☒	☐ MRI-based neuroimaging
